# Supplementary material for: Cell Fate Programming by Transcription Factors and Epigenetic Machinery in Stomatal Development
Source: bioRxiv. 2023 Aug 24:2023.08.23.554515. Preprint. [Version 1] doi: 10.1101/2023.08.23.554515 (PMC10473704; doi:10.1101/2023.08.23.554515)
Supplement: 1 [file NIHPP2023.08.23.554515v1-supplement-1.pdf]

bioRxiv preprint doi: https://doi.org/10.1101/2023.08.23.554515; this version posted August 24, 2023. The copyright holder for this preprint (which was not certified by peer review) is the author/funder, who has granted bioRxiv a license to display the preprint in perpetuity. It is made available under aCC-BY 4.0 International license.

Supplementary Material for:

Cell Fate Programming by Transcription Factors and Epigenetic Machinery in Stomatal Development

Ao Liu[1*], Andrea Mair,[1*] Juliana L. Matos[2,3], Macy Vollbrecht[2], Shouling Xu[4], Dominique C. Bergmann[1,2]

[1] Howard Hughes Medical Institute, Stanford, CA, USA 94305
[2] Department of Biology, Stanford University, Stanford, CA, USA 94305
[3] Current address: Department of Chemical and Biomolecular Engineering, University of California, Berkeley, CA, USA 94720
[4] Carnegie Institution for Science, Stanford, CA, USA 94305
[*] : equal contribution

Supplemental Figures 1-9

Supplemental Table 1: Datasets analyzed in this work

Supplemental Table 2: List of primers

bioRxiv preprint doi: https://doi.org/10.1101/2023.08.23.554515; this version posted August 24, 2023. The copyright holder for this preprint (which was not certified by peer review) is the author/funder, who has granted bioRxiv a license to display the preprint in perpetuity. It is made available under aCC-BY 4.0 International license.

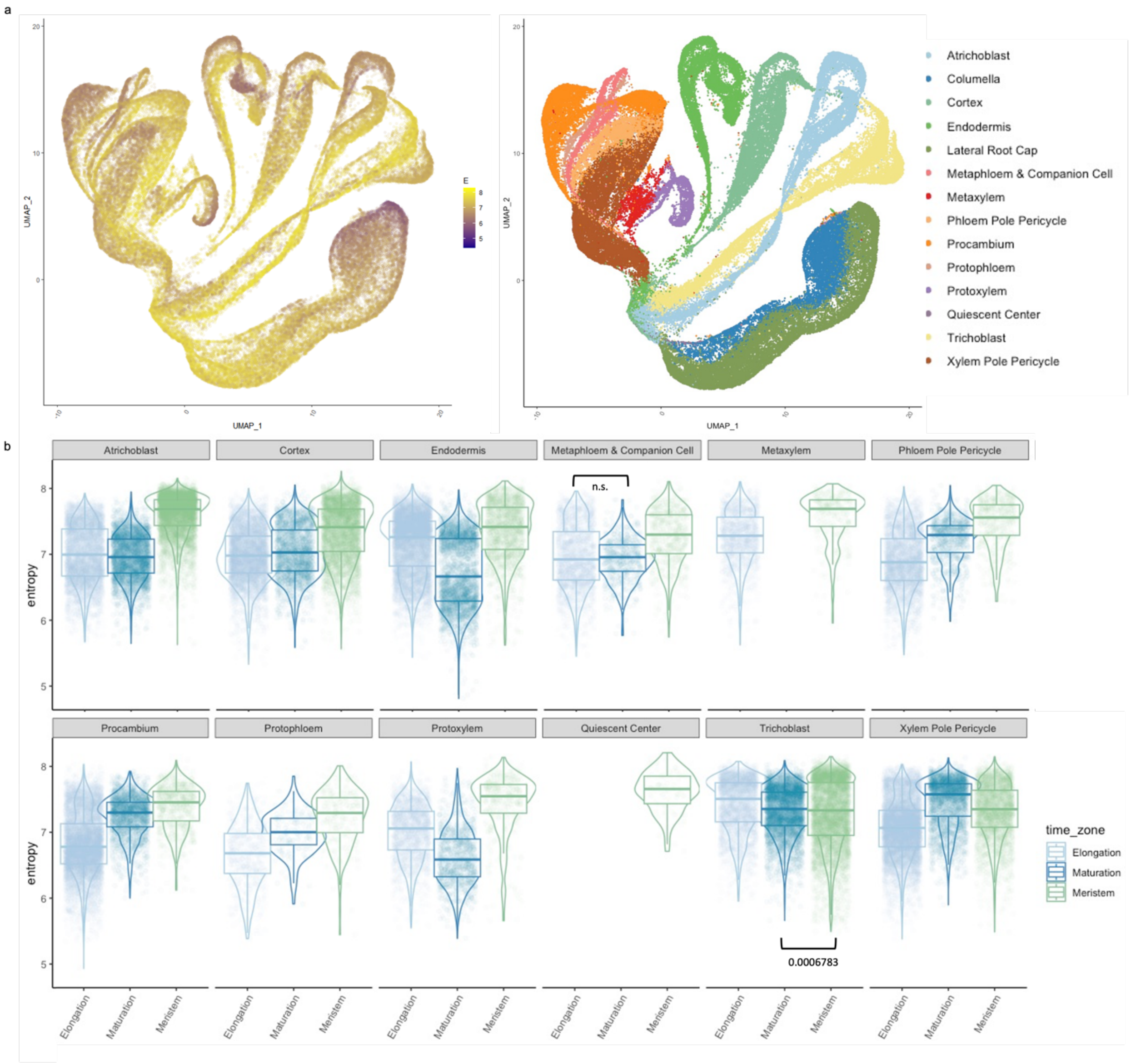

Supplemental Fig 1: **Transcriptional entropy during differentiation of root cell types**. **a.** Entropy scores (left) and cell identities (right) in root cells derived from scRNA-seq dataset in Shahan et al., [26]. **b.** Boxplot of entropy scores of different root cell types along a developmental gradient where meristem represents the least differentiated cells, followed by maturation and then elongation (most differentiated).

bioRxiv preprint doi: https://doi.org/10.1101/2023.08.23.554515; this version posted August 24, 2023. The copyright holder for this preprint (which was not certified by peer review) is the author/funder, who has granted bioRxiv a license to display the preprint in perpetuity. It is made available under aCC-BY 4.0 International license.

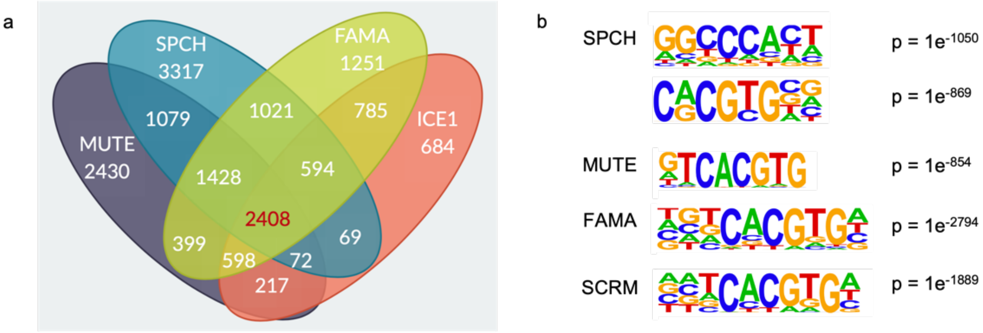

Supplemental Fig 2: **Shared properties among the stomatal lineage bHLHs' targets**. **a.** A Venn diagram showing the overlap of ChIP-seq peaks among the stomatal bHLHs, data sources shown in Table S1. **b.** Top motifs in the stomatal bHLHs' binding sites as determined by ChIP-seq of the respective bHLHs. Note that all factors recognize a G-box motif, but SPCH has an additional alternative preferred binding site.

bioRxiv preprint doi: https://doi.org/10.1101/2023.08.23.554515; this version posted August 24, 2023. The copyright holder for this preprint (which was not certified by peer review) is the author/funder, who has granted bioRxiv a license to display the preprint in perpetuity. It is made available under aCC-BY 4.0 International license.

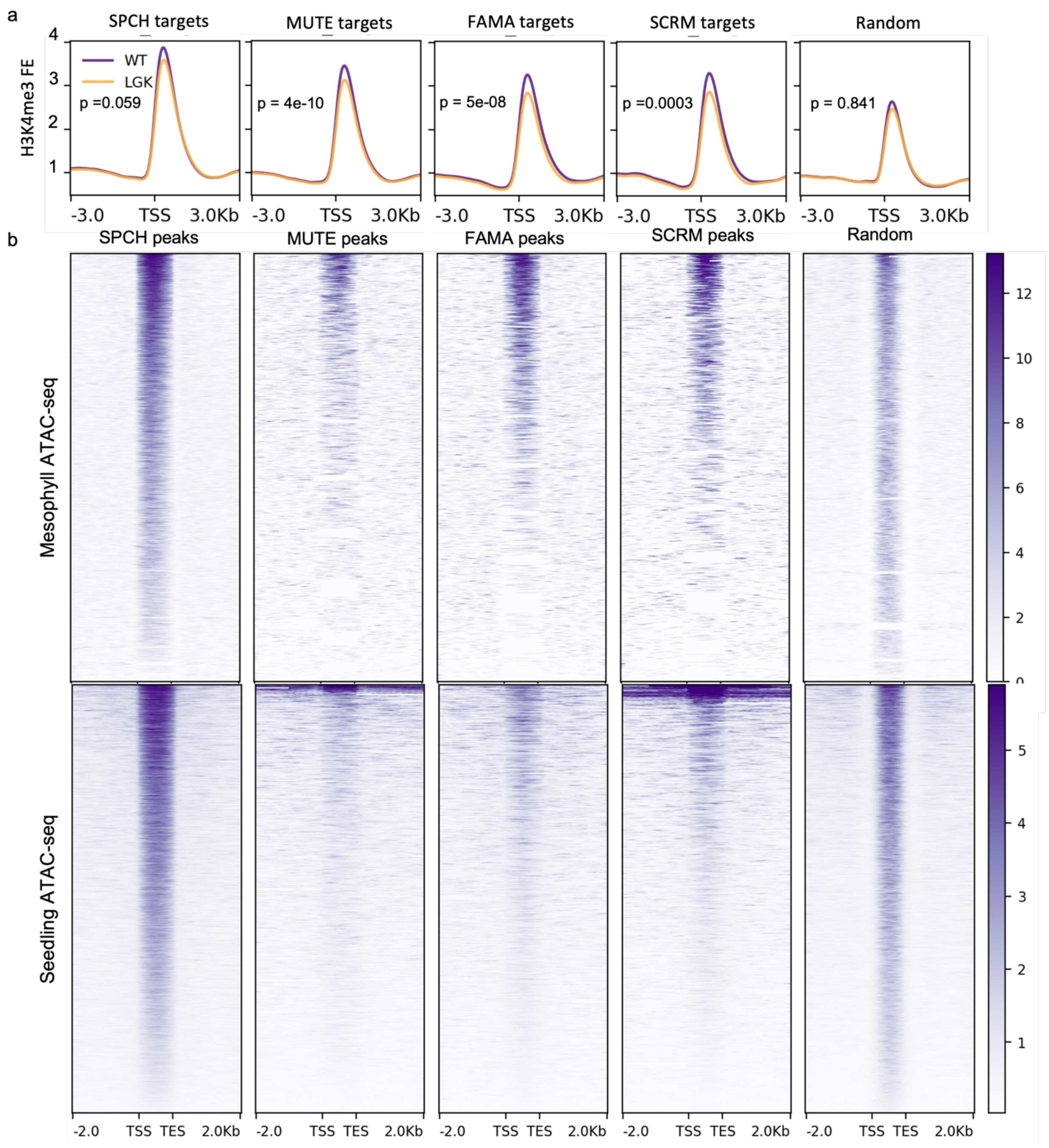

Supplemental Fig 3: **Chromatin landscapes at SPCH, MUTE, FAMA and SCRM targets**. **A.** Fold-enrichment (FE) of H3K4me3 levels in WT GC (purple) and FAMA^{LGK} GCs[37] ("pre-GCs" in orange) at targets of indicated stomatal bHLHs. **b.** Chromatin accessibility of stomatal bHLHs' binding sites and randomly sampled genomic intervals in mesophyll cells (top) and in whole seedlings (bottom)[34].

bioRxiv preprint doi: https://doi.org/10.1101/2023.08.23.554515; this version posted August 24, 2023. The copyright holder for this preprint (which was not certified by peer review) is the author/funder, who has granted bioRxiv a license to display the preprint in perpetuity. It is made available under aCC-BY 4.0 International license.

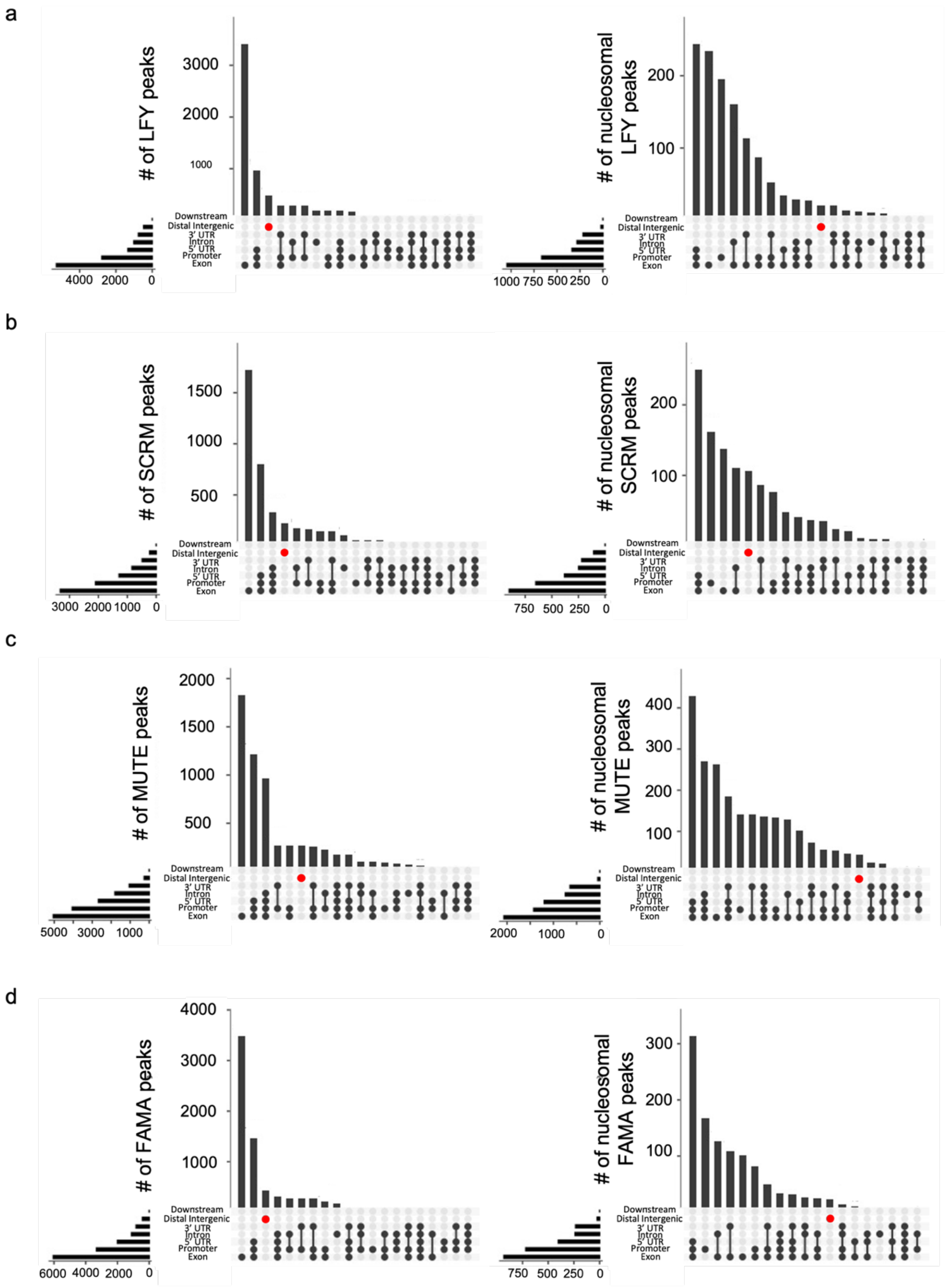

Supplemental Fig 4: **Nucleosomal binding sites of plant TFs are enriched in the gene body**. Histograms showing the distribution of all targets (left) and nucleosomal targets (right) of LFY (**a**), SCRM (**b**), MUTE (**c**) and FAMA (**d**). The red dot marks distal intergenic regions.

bioRxiv preprint doi: https://doi.org/10.1101/2023.08.23.554515; this version posted August 24, 2023. The copyright holder for this preprint (which was not certified by peer review) is the author/funder, who has granted bioRxiv a license to display the preprint in perpetuity. It is made available under aCC-BY 4.0 International license.

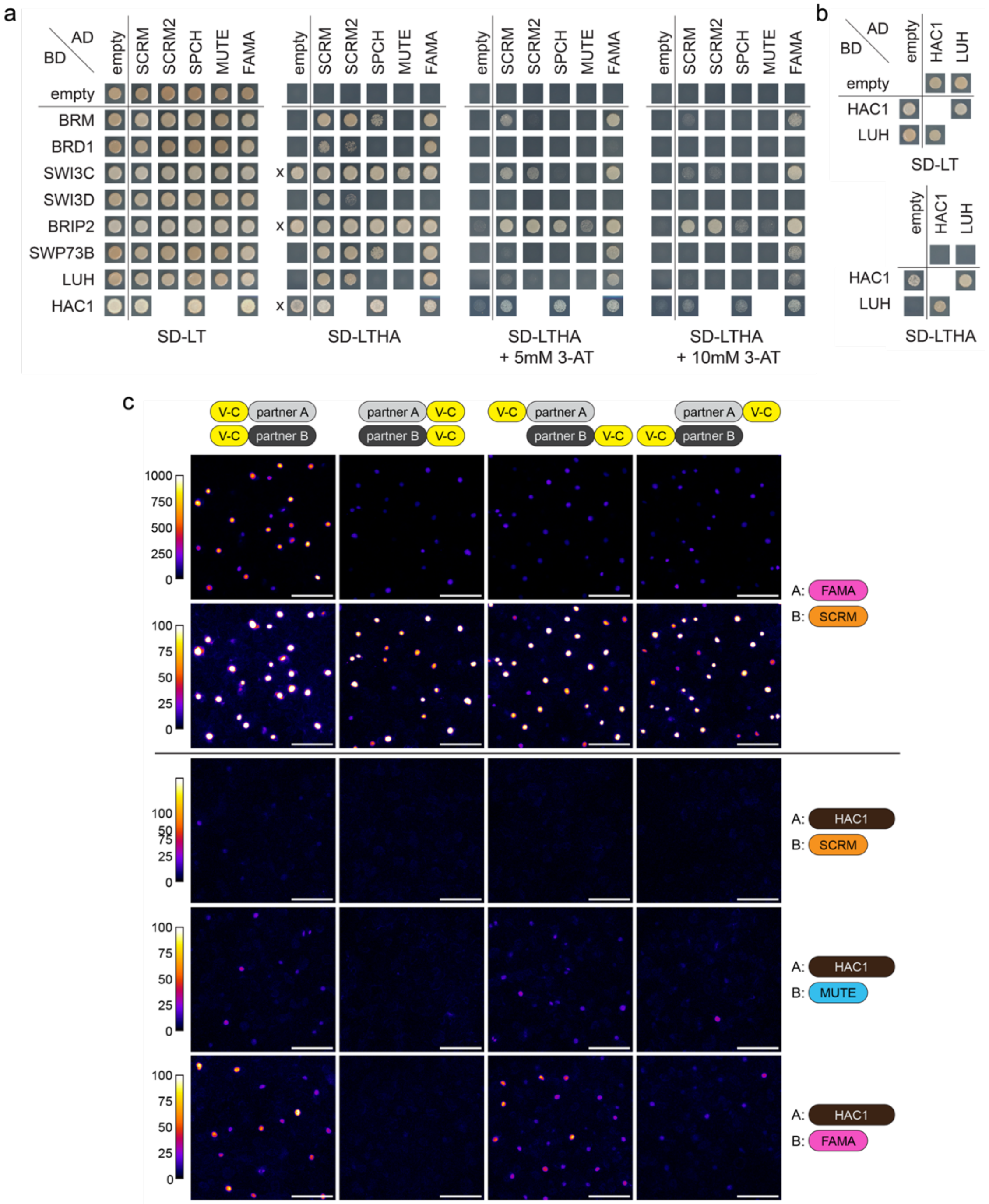

Supplemental fig 5: **Interaction between chromatin factors and stomatal lineage bHLHs**. **a.** Full panel of Y2H assays between activation domain (AD)-fused SCRM, SCRM2, SPCH, MUTE and FAMA and binding domain (BD)-fused SWI/SNF components and HAC1. Yeast was spotted onto SD-LT to confirm co-transformation of the constructs and onto SD-LTHA to test for interaction. 3-AT was added to some SD-LTHA plates as indicated to overcome auto-activation of SWI3C, BRIP2 and HAC1 (indicated by x). Interactions with SCRM are also shown in Fig 3b. **b.** Y2H of HAC1 and LUH. **c.** FAMA and MUTE, but not SCRM, interact weakly with HAC1 via bimolecular fluorescence complementation (BiFC) in *N. benthamiana*. V-N: Venus N-terminal half, V-C: Venus C-terminal half.

bioRxiv preprint doi: https://doi.org/10.1101/2023.08.23.554515; this version posted August 24, 2023. The copyright holder for this preprint (which was not certified by peer review) is the author/funder, who has granted bioRxiv a license to display the preprint in perpetuity. It is made available under aCC-BY 4.0 International license.

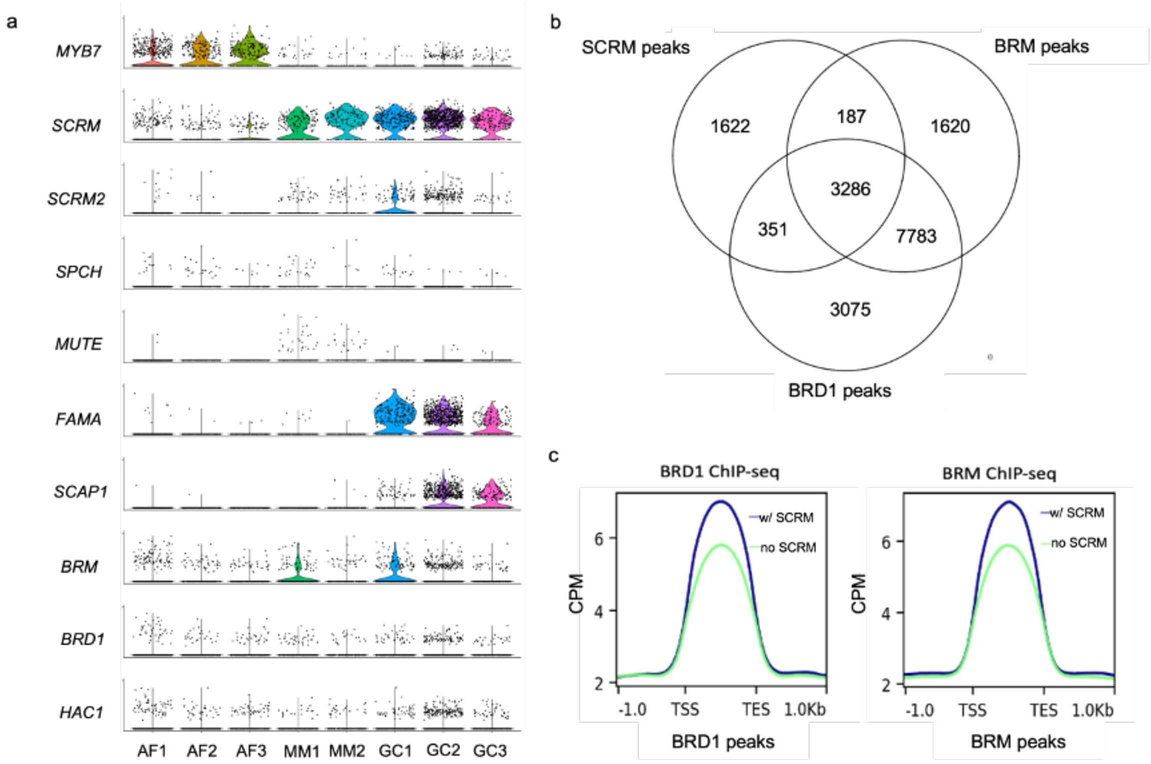

Supplemental Fig 6: ***BRM*, *BRD1* and *HAC1* are expressed in the stomatal lineage and the proteins bind to regions in the genome shared with the stomatal lineage bHLHs. a.** Expression levels of *BRM, BRD1, HAC1*, and stomatal lineage markers in the scRNA-seq dataset from young developing true leaves. Cell type abbreviations are AF: alternative epidermal (likely pavement cell) fate; MM: meristemoid; GC: Guard Cell. **b.** A Venn diagram showing the overlap of ChIP-seq peaks of BRM, BRD1 and SCRM. Data sources indicated in Table S1. **c.** Average ChIP-seq signal (Count Per Million Reads) at BRD1 and BRM peaks that overlap with SCRM (blue) and those that do not (green).

bioRxiv preprint doi: https://doi.org/10.1101/2023.08.23.554515; this version posted August 24, 2023. The copyright holder for this preprint (which was not certified by peer review) is the author/funder, who has granted bioRxiv a license to display the preprint in perpetuity. It is made available under aCC-BY 4.0 International license.

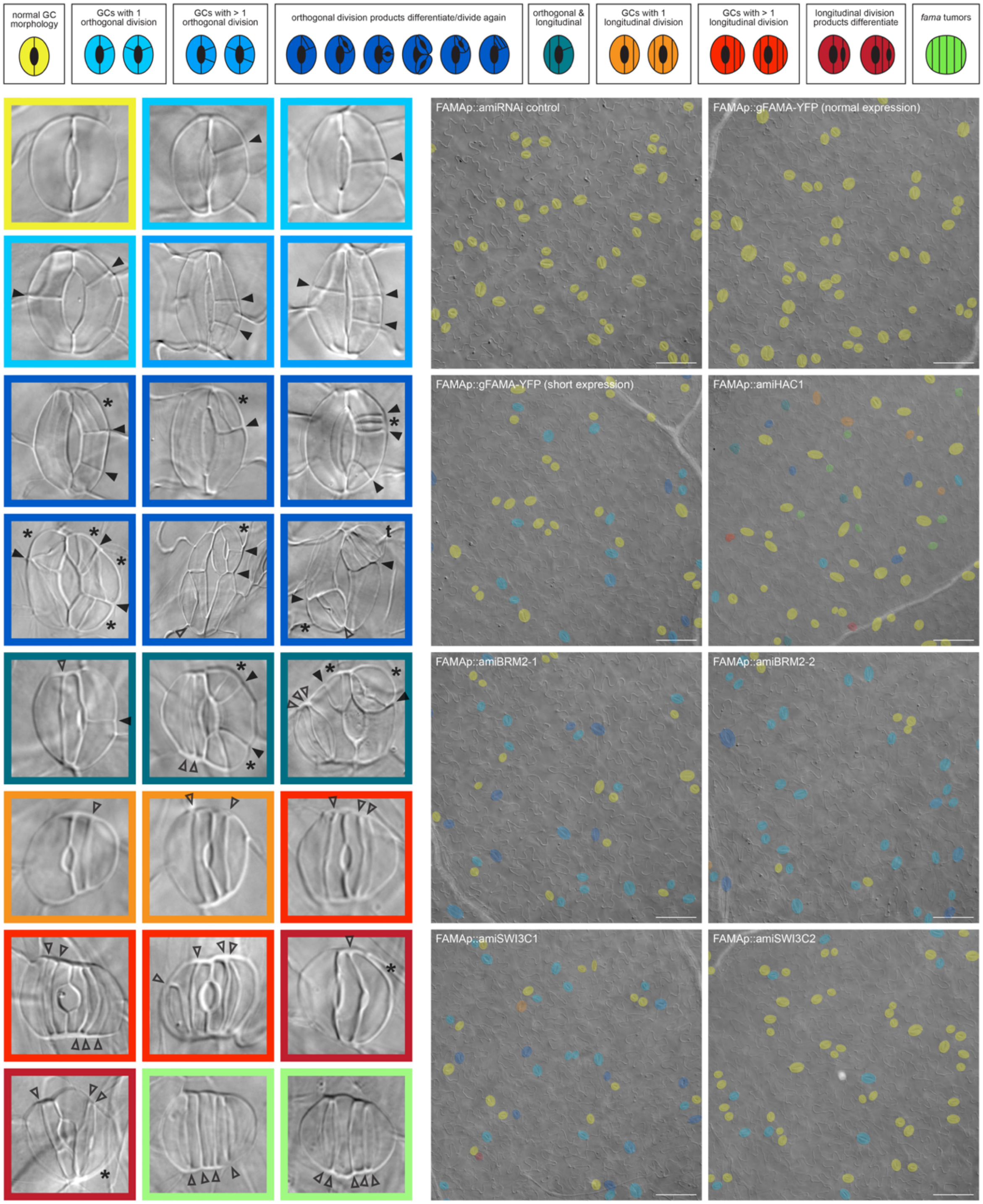

Supplemental Fig 7: ***FAMAp::amiRNA* lines display diverse developmental defects in GCs. Top**: GC variations observed in the lines described in Fig 5a are shown as simplified schemes in the top panel. **Left**: Examples for each class of GCs image borders are color-coded to match the top scheme. Transverse and longitudinal extra divisions in GCs are marked by filled and unfilled arrowheads, respectively. GCs within GCs are marked by an asterisk. **Right**: Zoomed out example images of the abaxial epidermis of the indicated lines showing the distribution of GCs with normal and abnormal morphology (overlays using the same color code as the top scheme). The images are DIC images of cleared 21 d old cotyledons. Scale bar = 100 µm.

bioRxiv preprint doi: https://doi.org/10.1101/2023.08.23.554515; this version posted August 24, 2023. The copyright holder for this preprint (which was not certified by peer review) is the author/funder, who has granted bioRxiv a license to display the preprint in perpetuity. It is made available under aCC-BY 4.0 International license.

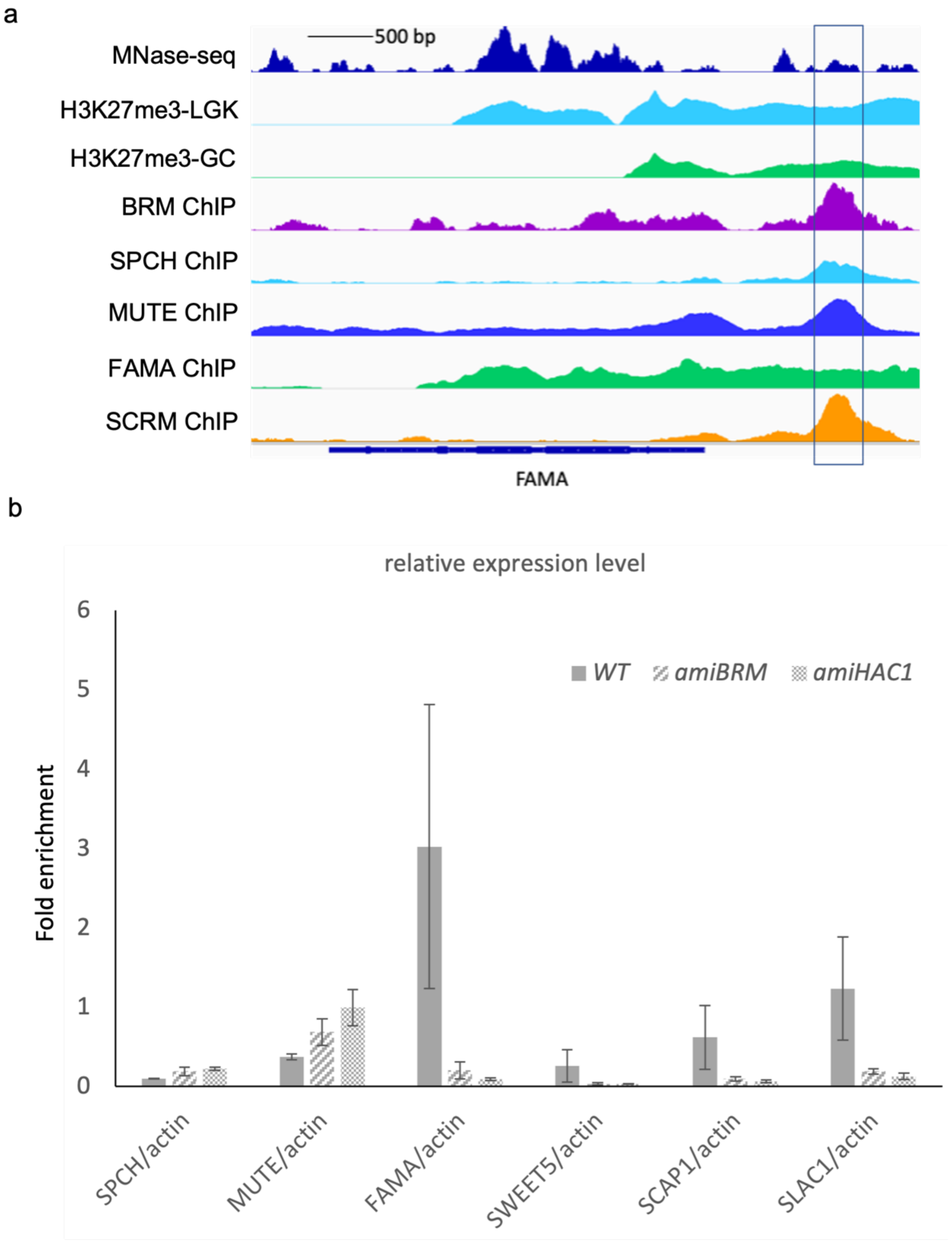

Supplemental Fig 8: ***FAMAp::amiRNA* lines fail to upregulate FAMA targets. a.** View of the FAMA genomic locus, repressive H3K27me3 signal in “pre-GCs” (LGK, light blue) and GCs (green) and binding of BRM (purple) and the stomatal bHLHs (SPCH, light blue; MUTE, dark blue; FAMA, green; SCRM, orange) from ChIP-seq experiments. See Table S1 for data sources. **b.** Relative expression levels of SCRM-FAMA targets in *FAMAp::amiBRM* and *FAMApro::amiHAC1* lines as assayed by qRT-PCR; RNA extracted from 14 day old leaves and normalized to actin.

bioRxiv preprint doi: https://doi.org/10.1101/2023.08.23.554515; this version posted August 24, 2023. The copyright holder for this preprint (which was not certified by peer review) is the author/funder, who has granted bioRxiv a license to display the preprint in perpetuity. It is made available under aCC-BY 4.0 International license.

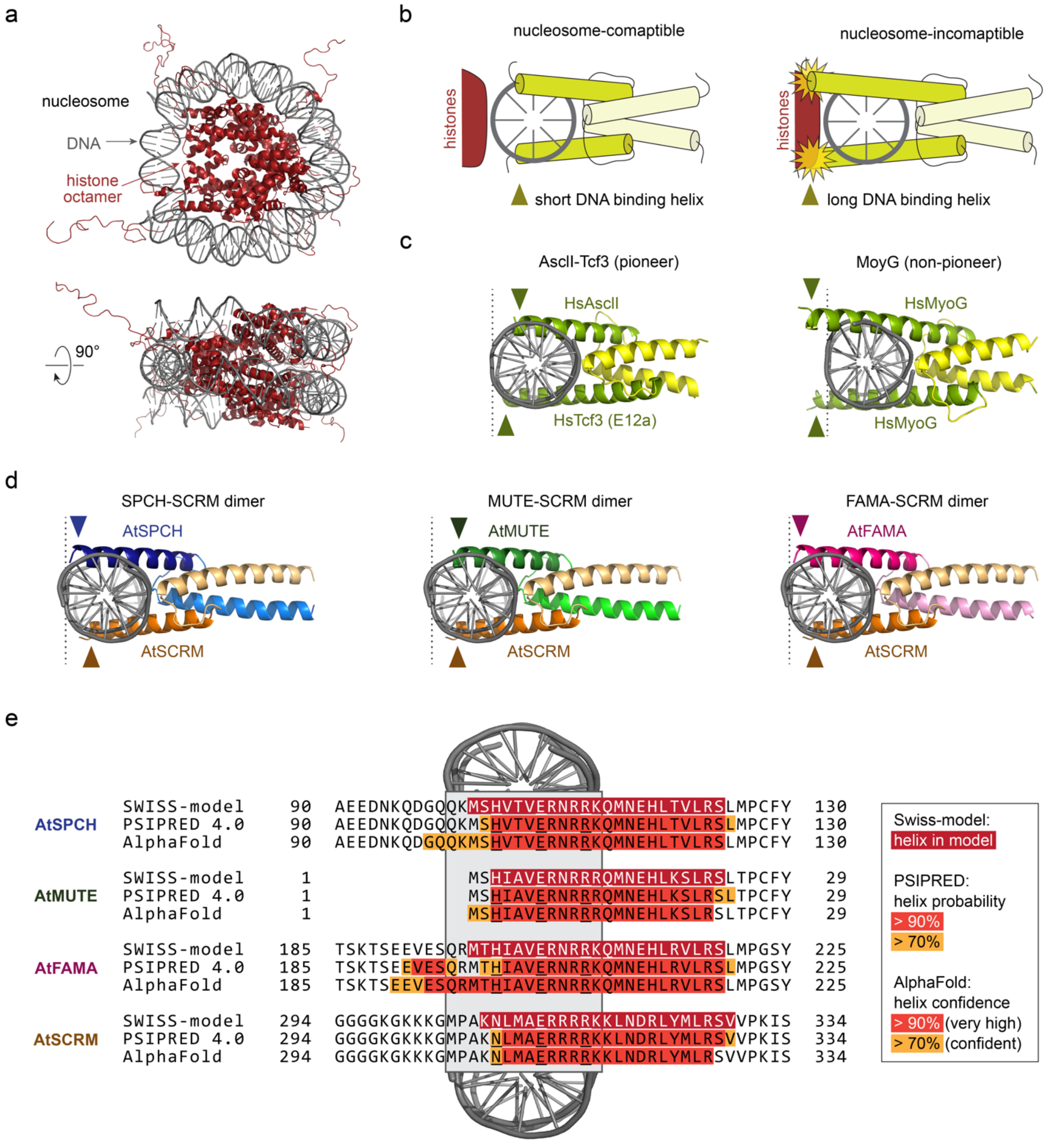

Supplemental Fig 9 **Predicted structure of DNA-bound SCRM dimers is compatible with nucleosome binding**. **a.** X-ray crystal structure of the nucleosome core particle at 1.9 A resolution (PDB: 1kx5). The DNA and histone octamer are shown in grey and dark red, respectively. **b.** Scheme of nucleosome-compatible and -incompatible bHLH dimers. Histones do not interfere with binding of short DNA binding helices on the other face of the DNA (left), while binding of long DNA binding helices is obstructed (right). **c.** Examples of human pioneer (Ascl1-Tcf3/E12a, left) and non-pioneer (MyoG, right) bHLH dimers, modelled with SWISS-model. The end of the DNA binding helix (dark green) is marked with an arrowhead. **d.** 3D structures of SCRM heterodimers modelled with SWISS-model. The end of the DNA helices is of similar length to the Ascl1-Tcf3

bioRxiv preprint doi: https://doi.org/10.1101/2023.08.23.554515; this version posted August 24, 2023. The copyright holder for this preprint (which was not certified by peer review) is the author/funder, who has granted bioRxiv a license to display the preprint in perpetuity. It is made available under aCC-BY 4.0 International license.

dimer and does not protrude beyond the DNA. **e.** Comparison of the length of the DNA binding helix in the models shown in (D) with additional structure predictions obtained with PSIPRED 4.0 and AlphaFold. Residues predicted to be part of the helix are highlighted in dark red (model), red (high confidence) and orange (medium confidence). The part of the helix that aligns with the DNA dimer is marked by a grey box. The three main residues that make contact with the DNA are underlined. Helix predictions for SCRM and MUTE are consistent between the three methods and always short. AlphaFold predicts an extension of the DNA-binding helix for SPCH and FAMA, which could interfere with nucleosome binding. Interestingly, in the PSIPRED prediction, this extension of the FAMA helix is interrupted by a stretch with very low helix probability, which could manifest as a flexible loop that breaks the rigid helix, thereby allowing nucleosome binding.

bioRxiv preprint doi: https://doi.org/10.1101/2023.08.23.554515; this version posted August 24, 2023. The copyright holder for this preprint (which was not certified by peer review) is the author/funder, who has granted bioRxiv a license to display the preprint in perpetuity. It is made available under aCC-BY 4.0 International license.

Supplemental Table 1: Datasets analyzed in this work

Dataset	Reference	PMID/PMCID	GEO #
Leaf epidermis scRNA-seq	Lopez-Anido et al., 2021	PMC8054824	GSE167135
Root scRNA-seq	Shahan et al., 2022	PMC9014886	GSE152766
SPCH ChIP-seq	Lau et al., 2014	PMC4390554	GSE57954
MUTE ChIP-seq	Han et al., 2018	PMID: 29738710	GSE107018
SCRM ChIP-seq	Tang et al., 2020	PMID: 32068336	GSE145258
BRM ChIP-seq	Yu et al., 2019	PMID: 33771698	GSE142369
BRD1 ChIP-seq	Yu et al., 2021	PMID: 33771698	GSE161595
LFY ChIP-seq	Jin et al., 2021	PMC7840934	GSE141706
PIF4 ChIP-seq	Oh et al., 2012	PMC3703456	GSE35315
MNase-seq	Chodavarapu et al., 2010	PMC2964354	GSE21673
INTACT ATAC-seq dataset 1	Kim et al., 2022	PMC9788986	GSE190753
INTACT ATAC-seq dataset 2	Lu et al., 2017	PMC5389718	GSE85203
H3K27me3 ChIP-seq in GCs	Lee et al., 2019	PMC6815143	GSE118138
H3K27me3 ChIP-seq in $FAMA^{LGK}$ GCs	Lee et al., 2019	PMC6815143	GSE118138
FAMA Chip-seq data	This work		GSE*XXXXXX*
Proteomics data	This work		PRIDE dataset identifier PXD040690

bioRxiv preprint doi: https://doi.org/10.1101/2023.08.23.554515; this version posted August 24, 2023. The copyright holder for this preprint (which was not certified by peer review) is the author/funder, who has granted bioRxiv a license to display the preprint in perpetuity. It is made available under aCC-BY 4.0 International license.

Supplemental Table 2: primer list

primer	experiment	sequence
SCRMp fw	PL	ACCACCGTCAATAACATCGTTAAGTAG
SCRMp rv	PL	CGCCAAAGTTGACACCTTTACCCC
SCRM no stop fw	PL, BiFC	CACCATGGGTCTTGACGGAAACAATGG
SCRM no stop rv	PL, BiFC	GATCATACCAGCATACCCTGCT
SCRM2 Y2H fw	Y2H	ACCAGAATTCATGAACAGCGACGGTGTTTGGC
SCRM2 Y2H rev	Y2H	CAAGGATCCTCAAACCAAACCAGCGTAACCTGCT
SPCH Y2H fw	Y2H	GTGAATTCATGCAGGAGATAATACC
SPCH Y2H rev	Y2H	GTGGATCCCTAGCAGAATGTTTGC
MUTE Y2H fw	Y2H	GTGAATTCATGTCTCACATCGCTG
MUTE Y2H rev	Y2H	TGGATCCTTAATTGGTAGAGACGATC
FAMA Y2H fw	Y2H	ACCAGAATTCATGGATAAAGATTACTCGGCACCAAACT
FAMA Y2H rev	Y2H	CAAGGATCCTCAAGTAAACACAATATTTCCCAGGTTAGAGC
BRM Y2H fw	Y2H	CACCATGCAATCTGGAGGCAGTGGC
BRM Y2H rev	Y2H	CAAGGATCCCTATAAATGGCTAGGCCGTCTTTTAC
BRD1 Y2H fw	Y2H	CACCATGGGTGAGGTAGCAGATACA
BRD1 Y2H rev	Y2H	CAGCTGTAATGCCAGATCCG
SWI3C Y2H fw	Y2H	CACCATGCCAGCTTCTGAAGATAGAAGAG
SWI3C Y2H rev	Y2H	CAAGGATCCTTAGTTTAAGCCTAAGCCGGACC
SWI3D Y2H fw	Y2H	CACCATGGAGGAAAAACGACGCGATTC
SWI3D Y2H rev	Y2H	CAAGGATCCCTAAACCGAAGAAACATTGTCTGAAC
BRIP2 Y2H fw	Y2H	CACCATGGAGGACACGAAGCCA
BRIP2 Y2H rev	Y2H	CCGGCTATTCAAATCAAATTCTCC
SWP73B Y2H fw	Y2H	CACCATGTCTGGTAACAACAACAATCC
SWP73 Y2H rev	Y2H	CCAACTCCCTGGCCCA
LUH Y2H fw	Y2H	CACCATGGCTCAGAGTAATTGGGAAG
LUH Y2H rev	Y2H	CTTCCAAATCTTTACGGATTTGTCATG
HAC1 fw	Y2H, BiFC	CACCATGAATGTTCAGGCTCACATGTCG
HAC1 stop rev	Y2H, BiFC	GAAGATCAGCCAACCAAACCCC
HAC1 no stop rev	BiFC	ACCTGAGCCCCCAGCGAC
SCRM stop BiFC rv	BiFC	TCAGATCATACCAGCATACCCTGCT
MUTE BiFC fw	BiFC	CACCATGTCTCACATCGCTGTTGAAAGGAATCG
MUTE nostop BiFC rv	BiFC	ATTGGTAGAGACGATCACTTCATCAGAC
MUTE stop BiFC rv	BiFC	TTAATTGGTAGAGACGATCACTTCATCA
FAMA BiFC fw	BiFC	CACCATGGATAAAGATTACTCGGCACCAAA
FAMA nostop BiFC rv	BiFC	TCAAGTAAACACAATATTTCCCAGGTTAG
FAMA stop BiFC rv	BiFC	AGTAAACACAATATTTCCCAGGTTAGAGC
amiHAC1 1-I	amiRNA	gaTACATTTATCACATTGGACCCtctctcttttgtattcc
amiHAC1 1-II	amiRNA	gaGGGTCCAATGTGATAAATGTAtcaaagagaatcaatga
amiHAC1 1-III	amiRNA	gaGGATCCAATGTGAAAAATGTTtcacaggtcgtgatatg
amiHAC1 1-IV	amiRNA	gaAACATTTTTCACATTGGATCCtctacatatatattcct
amiHAC1 2-I	amiRNA	gaTTTATCACATTGGACCCACGAtctctcttttgtattcc
amiHAC1 2-II	amiRNA	gaTCGTGGGTCCAATGTGATAAAtcaaagagaatcaatga
amiHAC1 2-III	amiRNA	gaTCATGGGTCCAATCTGATAATtcacaggtcgtgatatg
amiHAC1 2-IV	amiRNA	gaATTATCAGATTGGACCCATGAtctacatatatattcct
amiHAC1 3-I	amiRNA	gaTAGCAATTTTAAACAGGCCCTtctctcttttgtattcc
amiHAC1 3-II	amiRNA	gaAGGGCCTGTTTAAAATTGCTAtcaaagagaatcaatga
amiHAC1 3-III	amiRNA	gaAGAGCCTGTTTAATATTGCTTtcacaggtcgtgatatg
amiHAC1 3-IV	amiRNA	gaAAGCAATATTAAACAGGCTCTtctacatatatattcct
amiHAC1 4-I	amiRNA	gaTACCTATAATTGAGCCTGCAGtctctcttttgtattcc
amiHAC1 4-II	amiRNA	gaCTGCAGGCTCAATTATAGGTAtcaaagagaatcaatga

bioRxiv preprint doi: https://doi.org/10.1101/2023.08.23.554515; this version posted August 24, 2023. The copyright holder for this preprint (which was not certified by peer review) is the author/funder, who has granted bioRxiv a license to display the preprint in perpetuity. It is made available under aCC-BY 4.0 International license.

amiHAC1 4-III	amiRNA	gaCTACAGGCTCAATAATAGGTTtcacaggtcgtgatatg
amiHAC1 4-IV	amiRNA	gaAACCTATTATTGAGCCTGTAGtctacatatatattcct
amiBRM 1-I	amiRNA	gaTACAAATTTGCGGTACGCCCTtctctcttttgtattcc
amiBRM 1-II	amiRNA	gaAGGGCGTACCGCAAATTTGTAtcaaagagaatcaatga
amiBRM 1-III	amiRNA	gaAGAGCGTACCGCATATTTGTTtcacaggtcgtgatatg
amiBRM 1-IV	amiRNA	gaAACAAATATGCGGTACGCTCTtctacatatatattcct
amiBRM 2-I	amiRNA	gaTACTCTAGTCTTTCTACGCGGtctctcttttgtattcc
amiBRM 2-II	amiRNA	gaCCGCGTAGAAAGACTAGAGTAtcaaagagaatcaatga
amiBRM 2-III	amiRNA	gaCCACGTAGAAAGAGTAGAGTTtcacaggtcgtgatatg
amiBRM 2-IV	amiRNA	gaAACTCTACTCTTTCTACGTGGtctacatatatattcct
amiSWI3C 1-I	amiRNA	gaTTAACGCTTGCAATTGCGCAAtctctcttttgtattcc
amiSWI3C 1-II	amiRNA	gaTTGCGCAATTGCAAGCGTTAAtcaaagagaatcaatga
amiSWI3C 1-III	amiRNA	gaTTACGCAATTGCATGCGTTATtcacaggtcgtgatatg
amiSWI3C 1-IV	amiRNA	gaATAACGCATGCAATTGCGTAAtctacatatatattcct
amiSWI3C 2-I	amiRNA	gaTGATTTCATCCTATCGTCCGCtctctcttttgtattcc
amiSWI3C 2-II	amiRNA	gaGCGGACGATAGGATGAAATCAtcaaagagaatcaatga
amiSWI3C 2-III	amiRNA	gaGCAGACGATAGGAAGAAATCTtcacaggtcgtgatatg
amiSWI3C 2-IV	amiRNA	gaAGATTTCTTCCTATCGTCTGCtctacatatatattcct
amiSCRAMBLE F	amiRNA	CACC CCCAAACACACGCTCGGACGC
amiSCRAMBLE R	amiRNA	GCCGCTCTAGAACTAGTGGATCC
FAMA qPCR F1	qPCR	ACAACAGCAGCAACATCAACT
FAMA qPCR R1	qPCR	GCATGAGATGTTGGGTTTGGT
FAMA qPCR F2	qPCR	CCAACGGATGACTCATATCGC
FAMA qPCR R2	qPCR	TCATGTCCCTACCGGTTTCTC
SCAP1 qPCR F1	qPCR	CAAACCAAGAAACCACCGTCT
SCAP1 qPCR R1	qPCR	ATGGGGACGTTTCTTAGAGCA
SCAP1 qPCR F2	qPCR	GAAACTGTTGTCGTCGAGAGG
SCAP1 qPCR R2	qPCR	TCACTTCCTCCTCCTCCTGTA
SLAC1 qPCR F1	qPCR	ACGCTCAGCAAACAAAAGTCT
SLAC1 qPCR R1	qPCR	CCTTAGGAGAAACGGCCATTG
SLAC1 qPCR F2	qPCR	GACCAAACCGAGGGAAACAAA
SLAC1 qPCR R2	qPCR	CTCCACCGTTGATGATTCCAC
SLAC1 qPCR F3	qPCR	GTTGTAGGGAATTTCGTCGGG
SLAC1 qPCR R3	qPCR	GGGGCAGCAATGAACATAGAG
ACT2 qPCR F1	qPCR	TGTCTCGTTGTCCTCCTCACT
ACT2 qPCR R1	qPCR	ACAAGATCGAGATCCAGCAAA
ACT2 qPCR F2	qPCR	GTTGGGATGAACCAGAAGGAT
ACT2 qPCR R2	qPCR	GAGGAGCCTCGGTAAGAAGAA
MUTE qPCR F1	qPCR	CACATCGCTGTTGAAAGGAAT
MUTE qPCR R1	qPCR	GTCGGTTTAGGGTCTTTCGAC
MUTE qPCR F2	qPCR	GTTGTCTCTAGGCGAATCGTG
MUTE qPCR R2	qPCR	TCAAGAGTTAGCTCCTCCAAGC
SPCH qPCR F1	qPCR	TGAAGGTGCCGGAGAGATATC
SPCH qPCR R1	qPCR	CCGTCTCCGTCTTCTTCTTCT
SPCH qPCR F2	qPCR	TCCCGGGACAAGTTATGAAGA
SPCH qPCR R2	qPCR	TGCTGAATTTGTTGAGCCAGT
SWEET5 qPCR F1	qPCR	TCCGAGTTTAAGCCAGATCCA
SWEET5 qPCR R1	qPCR	GCGAAGACAAAGAAGATGGTGA
SWEET5 qPCR F2	qPCR	CGTTTGGGTCATTTATGCATGTC
SWEET5 qPCR R2	qPCR	CTATCAAGCCTGGCCAAGTTC
